# A Mixed Methods Exploration of the Impact of the COVID-19 Pandemic on Food-Related Activities and Diet Quality in People with Parkinson Disease

**DOI:** 10.3390/ijerph191811741

**Published:** 2022-09-17

**Authors:** Christine C. Ferguson, Seung E. Jung, Jeannine C. Lawrence, Joy W. Douglas, Anne Halli-Tierney, Chuong Bui, Amy C. Ellis

**Affiliations:** 1 UAB/Lakeshore Research Collaborative, University of Alabama at Birmingham, Birmingham, AL 35294, USA; 2 Department of Human Nutrition and Hospitality Management, University of Alabama, Tuscaloosa, AL 35487, USA; 3 Department of Family, Internal, and Rural Medicine, University of Alabama, Tuscaloosa, AL 35487, USA; 4 Alabama Life Research Institute, University of Alabama, Tuscaloosa, AL 35487, USA

**Keywords:** Parkinson disease, food-related activities, qualitative, diet, healthy diet, diet survey, Mediterranean diet

## Abstract

Objective: The purpose of this mixed methods study was to explore the impact of COVID-19 on the ability of people with Parkinson disease (PwPs) and their care-partners to perform food-related activities (FRA) and PwPs’ overall diet quality. Methods: Using a convergent parallel mixed methods design, PwPs and their care-partners completed virtual dyadic semi-structured interviews about their FRA during the COVID-19 pandemic. PwPs completed Food Frequency Questionnaires (FFQ) to quantify their dietary intake in the previous 12 months. Qualitative data were analyzed by two coders using thematic analysis, and quantitative data from FFQs were descriptively analyzed to calculate diet quality scores. Results: Eleven dyadic interviews revealed the following key themes: cooking more at home; changes with grocery shopping; less meals with non-household members. These changes were described to increase the care-partners’ responsibilities and overall burden. Diet scores among PwPs were 73.0 ± 6.3 for the Healthy Eating Index 2015 (scale of 0–100), 29.2 ± 6.6 for the Mediterranean diet (scale of 0–55), and 10.4 ± 1.8 for the Mediterranean-DASH Intervention for Neurodegenerative Delay (MIND) diet (scale 0–15). Conclusions: Diet scores revealed that PwPs consumed a high-quality diet during the pandemic. Findings from this study highlight the need for tailored nutrition education to support PwPs’ care-partners.

## 1. Introduction

Parkinson disease (PD) is the second-most common neurological disease after dementia, and currently, 6.1 million people worldwide have been diagnosed with PD [1]. The risk of PD increases with age and primarily affects older adults of ages 60 and older [2]. PD is also considered a physical disability due to symptoms such as bradykinesia, tremors, freezing, and impaired gait, and people living with PD (PwPs) experience a multitude of non-motor symptoms such as depression, gastrointestinal hypomotility, dysphagia, and weight changes [3].

Disability-specific symptoms can negatively interfere with the ability of a person living with PD (PwP) to perform food-related activities (FRA), such as food preparation, grocery shopping, and eating [4,5,6]. Moreover, symptoms of PD can also increase their risk of malnutrition, which is further associated with a longer disease duration and greater symptom severity [7]. Community-dwelling older adults with PD may require assistance from a care-partner to perform activities of daily living, including FRA [8,9].

While there is no specific diet recommended for PwPs, emerging research supports the modification of dietary patterns to delay PD progression and cognitive decline. Based on observational data, it appears that both the Mediterranean and the Mediterranean DASH Intervention for Neurodegenerative Delay (MIND) diets may be beneficial to PwPs [10,11,12,13], but studies are lacking to describe the long-term effects of these dietary patterns in PD. Moreover, there is a need for a comprehensive investigation of the factors that may influence dietary quality in this population.

The World Health Organization formally declared a global pandemic due to coronavirus disease 2019 (COVID-19) on 11 March 2020 [14,15]. In efforts to reduce the transmission of the severe acute respiratory syndrome coronavirus 2 (SARS-COV-2), facemasks and social distancing were recommended [16]. Because older adults are at a higher risk for experiencing severe illness from COVID-19, these recommendations were even more strict for this population [17]. Many older adults with PD modified their daily activities during the COVID-19 pandemic to stay distanced from others, thereby remaining isolated at home [18]. Moreover, there is evidence that adults changed their dietary patterns during the pandemic with many consuming more processed foods and less fresh food products such as fruits and vegetables [19,20,21]. No studies have described the impact of the COVID-19 pandemic on diet quality and FRA in the PD population. Thus, the primary aims of this mixed methods study were to describe PwPs’ diet quality and how PwPs and their care-partners’ FRAs were impacted during the COVID-19 pandemic.

## 2. Materials and Methods

An exploratory, descriptive research design with semi-structured interviews was used for the qualitative arm of this study. PwPs’ diet quality was investigated using a validated food frequency questionnaire (FFQ) for the quantitative arm. Institutional Review Board approval was obtained, and data were collected between November 2020 and March 2021.

### 2.1. Participants

Through convenience sampling, PwPs and their care-partners were recruited to participate in this study. Sample size was determined based on qualitative data saturation [22]. The research team contacted PD support groups and organizations, both in person and online, to share the recruitment flyer. PwPs could participate if they had been diagnosed with PD, were 60 years old or older, lived in the United States, had access to the internet and Zoom, spoke English, and had a care-partner who was willing to join them for the qualitative interview. PwPs could not participate if they lived in a long-term care setting, including retirement housing, had cognitive impairment as assessed by the Short Portal Mental Status Questionnaire (SPMSQ) [23], or had aphasia or other severe speech and language impairment. There were no specific eligibility criteria for the care-partner.

Potential participants with PD were invited to complete a phone-screening interview to determine eligibility. Once a member of the research team determined they were eligible to participate, the participant reviewed the informed consent document and consented via email.

### 2.2. Interview Guide

The interview guide was developed with the intention of asking participants about any changes in their FRAs related to COVID-19 [6]. The semi-structured interview guide was critically assessed by the research team, which consisted of seven experts in nutrition, geriatrics, and qualitative methodology [24,25]. The interview guide was field-tested with a PwP and their care-partner. Based on results of the pilot testing, probing questions were added to improve comprehension, and the ordering of questions was reorganized for practicality. The questions about changes in FRA during the pandemic are presented in Table 1.

Dyadic interviews were scheduled during times convenient for the participants and their care-partners. All interviews were conducted via Zoom—A free videoconferencing webtool; additionally, interviews were video and audio recorded, transcribed verbatim, and checked for accuracy (Zoom Video Communications Inc., Banyai, Istvan, 2021). This study is part of a larger qualitative project with additional questions, and total interview lengths ranged from 27–83 min, with a mean average of 50 min [26].

The principal investigator (CF) interviewed participants about their FRAs and COVID-19. At the time of the study, the interviewer was a Registered Dietitian, had completed doctoral-level coursework in mixed methods research, and received qualitative research training to conduct individual interviews. A member of the research team (KO) reviewed the video recordings and took field notes to document any nonverbal or participant dynamic observations. Both the interviewer and research team member documented their overall impressions of each interview. These notes were used to support the interpretation of the responses. Two members of the research team (CF and AE) independently coded transcripts via thematic analysis using NVivo 12 software (QSR International, 2018, Melbourne, Australia). Any disagreements in coding were resolved through discussion, and a third team member (SJ) was available to make a coding decision as needed. Additional methods were used to improve trustworthiness of data such as member checking, triangulating data with field notes, and maintaining an audit trail.

### 2.3. Quantitative Data Collection and Analysis

Only PwPs completed the FFQ and disease severity assessment. PwPs completed the Diet History Questionnaire III (DHQ III)—A free, validated, 135-item FFQ that is self-administered electronically [27]. Diet scores can be calculated from FFQs to provide a metric of overall dietary patterns. DHQ III data were used to calculate the following diet scores: Healthy Eating Index 2015 (HEI-2015) [28] and Mediterranean diet [10], Mediterranean-DASH Intervention for Neurodegenerative Delay (MIND) diet scores [29]. The HEI-2015 represents the adherence to the 2015–2020 Dietary Guidelines for Americans (Supplemental Table S1) [28]. The Mediterranean diet and MIND diet scores represent adherence to the respective diets, and higher scores represent greater adherence to the dietary pattern (Supplemental Tables S2 and S3, respectively) [10,29].

Demographic data were collected during the phone screening, and disease severity was assessed using the Movement Disorder Society United Parkinson’s Disease Rating Scale (MDS-UPDRS) II [30]. PwPs completed the MDS-UPDRS II online via Qualtrics (Provo, UT, USA). Care-partners’ birth dates were obtained during the Zoom interview to calculate their age. Quantitative data were descriptively analyzed using SPSS 24 software (IBM, 2016, Armonk, NY, USA).

## 3. Results

Eleven dyadic interviews were conducted with PwPs and their care-partners. A total of 22 PwPs expressed interest in participating in the study. Seven did not meet the eligibility criteria, three declined due to time constraints, and one declined due to an acute medical issue. As shown in Table 2, the PwPs were 54.5% female, White (100%), and 72.7% college graduates. The mean PD duration was 10 years (SD 6.4, Range 0–26) and average disease severity score was 24.2 (SD 9.2, Range 14–42), which indicates moderate disability. Only one PwP reported a previous diagnosis of COVID-19. Diet score results are described in Table 3. A higher HEI score represents stronger adherence to the Dietary Guidelines for Americans, and higher Mediterranean and MIND diet scores indicate better adherence to those two dietary patterns. Through thematic content analysis, three themes were identified related to FRA and the COVID-19 pandemic and are summarized with participant identifiers in Table 4.

### 3.1. Theme A: Cooking More at Home

Participants shared how, due to the COVID-19 pandemic, the biggest change came from preparing their meals more at home rather than procuring meals from restaurant establishments. A care-partner stated, “Just cooking more, because as we said, we don’t eat out as often.” Participants stated they cooked more at home even with the option to pick meals up from restaurants. For example, one care-partner said, “But we, we still might grab something to go, but that, either. We don’t do that nearly as often as we did.”

Participants had varying opinions on whether this changed their overall dietary intake. One care-partner shared, “The only difference is that we eat in more. We don’t go out, but far as our daily diets, what we fix to eat, I don’t think it’s changed that much at all really.” A care-partner said that they have relied on eating more comfort food during the pandemic, and said, “I have to say that we probably have enjoyed more comfort foods of course. I’ve done more baking, lots of sweets, and I think that probably is something that we’re paying for. We were having less exercise because we’re going out less and eating more sweets and probably more chips and snack type foods as well.” Of those who consumed meals at restaurants prior to the pandemic, many reported that this change in their FRA have led to adopting healthier eating habits. Additionally, a component of the newer eating habits was reducing their intake of fried fast foods. A care-partner stated: “Well, I think, you asked about how a change since COVID. And I think what’s changed for us is mostly eating in, and eating in really has made us feel better. Even though we’ve been kind of closed in, not eating the run by and pick up fried chicken and French fries, and not run by and pick up a prefab something. Or something that’s been soaked in something, um, has made us- even though it’s not been an ideal situation by any stretch of the imagination and sometimes nerve racking- as far as food goes, I think we eat better than we did. Because we’re eating more at home, you know, things that we know what happened to ‘em before we got it.”

Participants stated that eating more meals at home meant that they spent more time on food preparation. This was identified as a problematic by care partners who are the primary meal preparers. They shared that it can be challenging to manage extra food preparation in addition to their other caregiving tasks. One care-partner said, “I guess sometimes there’s a lot of other responsibilities in his care. So sometimes it’s hard to get everything in with making larger food items. If I, if it wasn’t the pandemic that we, you know, would occasionally eat out more than we do. So, we’re doing a lot more food prep, so it’s more time consuming.”

### 3.2. Theme B: Changes with Grocery Shopping

Many participants described how the pandemic shifted the grocery shopping responsibilities, such that the care-partner took over as the primary shopper. Some PwPs were either the primary shoppers prior to the pandemic, or they shared that responsibility with their care-partner. Two participants shared how this changed because the care-partner felt more comfortable going grocery shopping than the PwPs. One said, “When COVID came along, it’s like, well, my system is probably not as hearty as his, so he’s the one who’s going to go out and do the grocery shopping and stuff.” Another PwP’s care-partner described how the PwP experienced anxiety when in public. “The difficulty might be, getting tired in the grocery store, because sometimes he’ll have to go to the car before I get finished. If there’s lots of people, the anxiety, you know, that kind of comes with too many people, especially during COVID getting around you. And I’ll say why don’t you just go to car and I’ll finish this, you know, whatever.”

Some participants reported less of a change with grocery shopping than others. Moreover, changes may have occurred early in the pandemic but had since reverted back to how they were prior to the pandemic. One PwP said, “Yeah. Initially, like you said, I wasn’t going to the stores at all. And then, you know, after three months or so I started going on occasion, and now I go weekly or a couple times a week.” Many participants reported trying grocery pickup options but ultimately preferring to purchase their own food. For example, a care-partner said, “When COVID first happened, we got a few, you know, curbside pickup, but now I go get them pretty much anytime I want you know, just precautionary measures and just go shopping, and we live close to several stores. That’s not an issue as far as driving.”

### 3.3. Theme C: Less Meals with Non-Household Members

Nearly every participant noted how they missed sharing meals with others as they did prior to the COVID-19 pandemic. For example, one participant with PD stated, “Well we used to have at least one or two or maybe three grandkids come every weekend before the pandemic and so I always have to cook for them… And now that with a pandemic they’re not coming over like that anymore.” Another said, “It cut out, pretty much all of our home entertainment, you know, like having people over, you know, enjoying a good meal with friends and family.”

Some adapted the way they spent time with others, such as eating outdoors while socially distanced. For example, one participant with PD shared, “We’ve had, I could probably count on one hand, the number of couples we’ve had over since COVID started, and in every instance, we ate outside on our patio.” Another shared a similar sentiment, “What we have done is, friends would say ‘let’s go out for dinner.’ Well instead of going out for dinner, go get what you want, in a carry out, and we’ll meet on our deck and eat or dine together. So that’s kind of been what we’ve done and that’s probably only done about three times this whole year.”

## 4. Discussion

Every participant reported a change in their typical FRA due to the COVID-19 pandemic. While the degree of change varied among participants based on their pre-pandemic habits, overall, they cooked more at home, consumed less meals with others, and modified their regular grocery shopping routine. Although cooking more meals at home can result in following a healthier dietary pattern, in this study, some care-partners reported that it was challenging to manage their other responsibilities since they were spending more time on food preparation and grocery shopping. Overall, PwPs’ diet scores reveal that they had a high diet quality during the pandemic. These findings highlight: (1) the increase in care-partner burden, (2) a decrease in social meals with others, and (3) the high diet quality PwPs exhibited during the COVID-19 pandemic.

The COVID-19 pandemic initiated mandatory changes in everyday life, and previous research has shown that the disease process of PD can make it more difficult for PwPs to adapt to changes, which can negatively impact mental health [31]. Participants reported anxiety and negative feelings related to going grocery shopping in public and the social isolation that resulted from stopping sharing meals with others. As a result, the care-partners, most of who were also older adults, typically took over the grocery shopping role. Our participants noted that this, in addition to cooking more at home, resulted in an increase in reported caregiver burden. This is a unique finding as a previous study reported the beneficial association from adults who cooked at home more during the pandemic [32]. Another study with older adults reported cooking as a form of entertainment and a stress reliever from the pandemic [33]. From the present study, the lack of socializing may have further increased care-partner stress as social isolation has been shown to be associated with nutrition risk for older adults [34]. Further research is needed to address caregiver burden of PwPs amidst the pandemic.

Our findings that PwPs and their care-partners cooked more at home due to the COVID-19 pandemic is consistent with the literature with adults of all ages [35,36,37,38,39,40]. Moreover, this observation is supported by a large, crowdsourced, cross-sectional study, the COVIDiet Study, that is investigating the dietary habits and quality of adults in 22 countries during the COVID-19 pandemic [39]. Five articles have been published thus far reporting a range of 29.9–62.1% of adults cooking more during the COVID-19 lockdown; however, this did not always correlate with a better adherence to the Mediterranean diet [35,37,38,39]. No study to date had examined the dietary changes of PwPs during the COVID-19 pandemic.

Previous studies reported that the general population tended towards unhealthier dietary practices during the COVID-19 pandemic, which was related to stress eating and coping with food [35,37,40]. Although few participants reported that they were eating more comfort foods than normal, the majority of our participants with PD said they maintained or improved their diet quality during the pandemic primarily due to positive care-partner support and successful coping strategies. Agarwal et al. suggested that the MIND diet may be the most beneficial in delaying the progression of PD when compared to the Mediterranean diet, and the most benefit was found in older adults with PD who had diet scores of 10.0 or higher [10]. Our participants’ mean MIND diet scores was 10.4 (range 8.0–13.5) Our participants also scored high for another relevant measure of diet quality, the HEI. Ranging from 0–100 with higher scores indicating better diet quality, our participants scored an average of 73.0 (range 66.8–82.0), and the average HEI score for Americans is 58 out of 100 [41]. Overall, our participants had a high diet quality during the COVID-19 pandemic.

Our participants found coping strategies to follow a healthy dietary pattern that may also be relevant post-pandemic. They described that they cooked more at home, avoided grocery shopping options where someone else outside the household chose their food products, such as with grocery store pickup or delivery, and brought their own foods when dining with others outdoors. Our study’s findings further emphasize the essential role care-partners serve in ensuring PwPs follow healthy dietary patterns, as they often assist the PwP with FRAs. Future research should investigate the experiences related to the FRAs and dietary intake of PwPs without care-partners to identify potential coping strategies, areas of assistance needed, and types of assistance received.

Several limitations should be taken into consideration when interpreting the results. The inherent limitations of convenience sampling and the collection of self-reported data can introduce sampling and reporting biases. Due to the pandemic, we could only include those who had access to the internet, which likely excluded PwPs who are of lower socioeconomic status, in rural areas, and/or have limited internet access. In efforts to make this study more accessible, we utilized Zoom, a free videoconferencing platform, that has been shown to be a cost-effective and user-friendly method of conducting qualitative interviews remotely [42]. As a result of the intention to limit care-partner participation burden, we collected limited demographic data of these participants. Future research focused on care-partners could expand these findings. Our participants with PD were White, well-educated, and supported by a care-partner; therefore, further research is warranted with a more diverse sample of PwPs, including those without care-partners. Most participants were considered moderately disabled due to PD, so additional research is needed to describe the experiences of PwPs with a greater disease severity.

## 5. Conclusions

To our knowledge, this is one of the first studies to describe the impact of the COVID-19 pandemic on PwPs’ FRAs and diet quality. Our participants shared how the pandemic prompted them to modify their regular cooking routines, shared meals with others, and grocery shopping habits. Overall, our sample of PwPs had a high diet quality, and some reported an improved diet due to the changes associated with the pandemic. While there were some positive perceptions of the health behaviors due to the pandemic, our results also highlighted how PwPs’ care-partners had an increase in burden due to more responsibilities with FRAs. Some participants reported how this increase in responsibility, including shopping for food, negatively impacted their mental health. Future research should investigate the long-term impacts of the COVID-19 pandemic on the dietary intake and habits of PwPs. Moreover, healthcare professionals and PD organizations should consider enhancing their efforts to provide food and nutrition-related support and mental health resources to care-partners during and after the pandemic. 

## Figures and Tables

**Table 1:** COVID-19 Interview Questions.

Since the pandemic, have your grocery shopping habits changed? How so? Since the pandemic, have your visits to restaurants changed? How so? Since the pandemic, have your food-related decisions changed? How so? Since the pandemic, have your cooking and meal preparation changed? How so? Are there other ways the pandemic has affected your food-related activities? How so?

**Table 2:** Characteristics of PwPs and care-partners.

Demographic	n	%	M ± SD	Range
**PwPs**				
Sex				
Male	5	45.5		
Female	6	54.5		
Age			65.8 ± 5.2	60–76
Ethnicity				
White	11	100		
PD duration, total			10.1 ± 6.4	0–26
Less than 5 years	1	9.1		
Between 5 and 10 years	6	54.5		
Greater than 10 years	4	36.4		
MDS-UPDRS II, total			24.2 ± 9.2	14–42
Mild disability	3	27.3		
Moderate disability	5	45.5		
Severe disability	3	27.3		
Education				
Some college	3	27.3		
College graduate	8	72.7		
History of COVID-19				
Yes	1	9.1		
No	10	90.9		
**Care-Partner**				
Age			66.9 ± 5.1	59–77

MDS-UPDRS II, Movement Disorder Society United Parkinson’s Disease Rating Scale Part II; PwPs, people living with Parkinson disease.

**Table 3:** PwPs’ diet score results.

Diet Score	M ± SD	Range	Maximum Possible Score
HEI	73.0 ± 6.3	66.8–82.0	100
Mediterranean	29.2 ± 6.6	22.0–45.0	55
MIND	10.4 ± 1.8	8.0–13.5	15

DASH, Dietary Approaches to Stop Hypertension; HEI, Healthy Eating Index; M, mean; MIND, Mediterranean-DASH Intervention for Neurodegenerative Delay; PwPs, people living with Parkinson disease; SD, standard deviation.

**Table 4:** Themes identified through dyadic interviews with PwPs and their care-partners about the impact of COVID-19 pandemic on food-related activities.

Themes	Demonstrative Quotes	
**A. Cooking more at home**	P4CP:	Just cooking more, because as we said, we don’t eat out as often.
	P11CP:	But we, we still might grab something to go, but that, either. We don’t do that nearly as often as we did.
	P1CP:	The only difference is that we eat in more, we don’t go out, but far as our daily diets, what we fix to eat, I don’t think it’s changed that much at all really.
	P7CP:	I guess sometimes there’s a lot of other responsibilities in his care. So sometimes it’s hard to get everything in with making larger food items. If I, if it wasn’t the pandemic that we, you know, would occasionally eat out more than we do. So, we’re doing a lot more food prep, so it’s more time consuming… I have to say that we probably have enjoyed more comfort foods of course. I’ve done more baking, lots of sweets, and I think that probably is something that we’re paying for. We were having less exercise because we’re going out less and eating more sweets and probably more chips and snack type foods as well.
	P2CP:	Well, I think, you asked about how a change since COVID. And I think what’s changed for us is mostly eating in, and eating in really has made us feel better. Even though we’ve been kind of closed in, not eating the run by and pick up fried chicken and French fries, and not run by and pick up a prefab something. Or something that’s been soaked in something, um, has made us- even though it’s not been an ideal situation by any stretch of the imagination and sometimes nerve racking- as far as food goes, I think we eat better than we did. Because we’re eating more at home, you know, things that we know what happened to ‘em before we got it.
**B. Changes with grocery shopping**	P3:	When COVID came along, it’s like, well, my system is probably not as hearty as his so he’s the one who’s going to go out and do the grocery shopping and stuff.
	P10:	Yeah. Initially, like you said, I was. I wasn’t going to the stores at all. And then, you know, after three months or so I started going on occasion, and now I go weekly or a couple times a week.
	P2CP:	The difficulty might be, getting tired in the grocery store, because sometimes he’ll have to go to the car before I get finished. If there’s lots of people, the anxiety, you know, that kind of comes with too many people, especially during COVID getting around you. And I’ll say why don’t you just go to car, and I’ll finish this, you know, whatever.
	P1CP:	When COVID first happened, we got a few, you know, curbside pickup, but now I go get them pretty much anytime I want you know, just precautionary measures and just go shopping, and we live close to several stores. That’s not an issue as far as driving.
**C. Less meals with non-household members**	P6:	Well we used to have at least one or two or maybe three grandkids come every weekend before the pandemic and so I always have to cook for them… And now that with a pandemic they’re not coming over like that anymore.
	P8:	It cut out, pretty much all of our home entertainment, you know, like having people over, you know, enjoying a good meal with friends and family.
	P3:	We’ve had, I could probably count on one hand, the number of couples we’ve had over since COVID started, and in every instance, we ate outside on our patio.
	P5:	What we have done is, friends would say ‘let’s go out for dinner.’ Well instead of going out for dinner, go get what you want, in a carry out, and we’ll meet on our deck and eat or dine together. So that’s kind of been what we’ve done and that’s probably only done about three times this whole year.

COVID-19, coronavirus disease 2019; PwPs, people living with Parkinson disease.

## Data Availability

Not applicable.

## Supplementary Materials

The following supporting information can be downloaded at: https://www.mdpi.com/article/10.3390/ijerph191811741/s1, Table S1: HEI-2015 components, point values, and standards for scoring; Table S2: Mediterranean diet score components, point values, and standards for scoring; Table S3: MIND diet score components, point values, and standards for scoring. References [10,28,29] are cited in the supplementary materials.

## References

[1] Hirsch L., Jette N., Frolkis A., Steeves T., Pringsheim T. (2016). The incidence of Parkinson’s disease: A systematic review and meta-analysis. Neuroepidemiology.

[2] Jankovic J. (2008). Parkinson’s disease: Clinical features and diagnosis. J. Neurol. Neurosurg. Psychiatry.

[3] Marcason W. (2009). What are the primary nutritional issues for a patient with Parkinson’s disease?. J. Am. Diet. Assoc..

[4] Vescovelli F., Sarti D., Ruini C. (2018). Subjective and psychological well-being in Parkinson’s Disease: A systematic review. Acta Neurol. Scand..

[5] Plastow N.A., Atwal A., Gilhooly M. (2015). Food activities and identity maintenance in old age: A systematic review and meta-synthesis. Aging Ment. Health.

[6] Sheard J.M., Ash S., Silburn P.A., Kerr G.K. (2011). Prevalence of malnutrition in Parkinson’s disease: A systematic review. Nutr. Rev..

[7] Bhimani R. (2014). Understanding the burden on caregivers of people with Parkinson’s: A scoping review of the literature. Rehabil. Res. Pract..

[8] Shin J.Y., Habermann B. (2020). Key activities of caregivers for individuals with Parkinson disease: A secondary analysis. J. Neurosci. Nurs..

[9] Agarwal P., Wang Y., Buchman A.S., Holland T.M., Bennett D.A., Morris M.C. (2018). MIND diet associated with reduced incidence and delayed progression of parkinsonism in old age. J. Nutr. Health Aging.

[10] Morris M.C., Tangney C.C., Wang Y., Sacks F.M., Barnes L.L., Bennett D.A., Aggarwal N.T. (2015). MIND diet slows cognitive decline with aging. Alzheimers Dement..

[11] Berendsen A.M., Kang J.H., Feskens E.J.M., de Groot C., Grodstein F., van de Rest O. (2018). Association of long-term adherence to the MIND diet with cognitive function and cognitive decline in American women. J. Nutr. Health Aging.

[12] Paknahad Z., Sheklabadi E., Derakhshan Y., Bagherniya M., Chitsaz A. (2020). The effect of the Mediterranean diet on cognitive function in patients with Parkinson’s disease: A randomized clinical controlled trial. Complement. Ther. Med..

[13] Shahid Z., Kalayanamitra R., McClafferty B., Kepko D., Ramgobin D., Patel R., Aggarwal C.S., Vunnam R., Sahu N., Jain R. (2020). COVID-19 and older adults: What we know. J. Am. Geriatr. Soc..

[14] Timeline: WHO’s COVID-19 Response. https://www.who.int/emergencies/diseases/novel-coronavirus-2019/interactive-timeline?gclid=CjwKCAjwqIiFBhAHEiwANg9szjjygqYGLAPIJb-brxm8uXhdOZHgcytMsyoPk4p85nIE_TqKVFs5IhoCaIsQAvD_BwE#event-12.2021.

[15] Moreland A., Herlihy C., Tynan M.A., Sunshine G., Mccord R.F., Hilton C., Poovey J., Werner A.K., Jones C.D., Fulmer E.B. (2020). Timing of state and territorial COVID-19 stay-at-home orders and changes in population movement—United States, March 1–May 31, 2020. MMWR Morb. Mortal. Wkly. Rep..

[16] COVID-19: Older Adults. Centers for Disease Control and Prevention. https://www.cdc.gov/coronavirus/2019-ncov/need-extra-precautions/older-adults.html.2021.

[17] Anghelescu B.A., Bruno V., Martino D., Roach P. (2021). Effects of the COVID-19 pandemic in Parkinson’s disease: A single-centered qualitative study. Can. J. Neurol. Sci..

[18] Jafri A., Mathe N., Aglago E.K., Konyole S.O., Ouedraogo M., Audain K., Zongo U., Laar A.K., Johnson J., Sanou D. (2021). Food availability, accessibility and dietary practices during the COVID-19 pandemic: A multi-country survey. Public Health Nutr..

[19] Di Santo S.G., Franchini F., Filiputti B., Martone A., Sannino S. (2020). The effects of COVID-19 and quarantine measures on the lifestyles and mental health of people over 60 at increased risk of dementia. Front. Psychiatry.

[20] Ammar A., Brach M., Trabelsi K., Chtourou H., Boukhris O., Masmoudi L., Bouaziz B., Bentlage E., How D., Ahmed M. (2020). Effects of COVID-19 home confinement on eating behaviour and physical activity: Results of the ECLB-COVID19 international online survey. Nutrients.

[21] Erkinjuntti T., Sulkava R., Wikstrom J., Autio L. (1987). Short Portable Mental Status Questionnaire as a screening test for dementia and delirium among the elderly. J. Am. Geriatr. Soc..

[22] Creswell J.W. (1998). Qualitative Inquiry and Research Design: Choosing among Five Traditions.

[23] Barriball K.L., While A. (1994). Collecting data using a semi-structured interview: A discussion paper. J. Adv. Nurs..

[24] Kallio H., Pietilä A.M., Johnson M., Kangasniemi M. (2016). Systematic methodological review: Developing a framework for a qualitative semi-structured interview guide. J. Adv. Nurs..

[25] (2018). Diet History Questionnaire, Version 3.0. National Institutes of Health, Epidemiology and Genomics Research Program.

[26] Ferguson C.F., Jung S.E., Lawrence J.C., Halli-Tierney A., Bui C., Ellis A.C. (2022). A qualitative analysis of experiences with food-related activities among people living with Parkinson disease and their care-partners. J. Appl. Gerontol..

[27] Krebs-Smith S.M., Pannucci T.E., Subar A.F., Kirkpatrick S.I., Lerman J.L., Tooze J.A., Wilson M.M., Reedy J. (2018). Update of the Healthy Eating Index: HEI-2015. J. Acad. Nutr. Diet..

[28] Panagiotakos D.B., Pitsavos C., Arvaniti F., Stefanadis C. (2007). Adherence to the Mediterranean food pattern predicts the prevalence of hypertension, hypercholesterolemia, diabetes and obesity, among healthy adults; the accuracy of the MedDietScore. Prev. Med..

[29] Goetz C.G., Fahn S., Martinez-Martin P., Poewe W., Sampaio C., Stebbins G.T., Stern M.B., Tilley B.C., Dodel R., Dubois B. (2007). Movement Disorder Society-sponsored revision of the Unified Parkinson’s Disease Rating Scale (MDS-UPDRS): Process, format, and clinimetric testing plan. Mov. Disord..

[30] Helmich R.C., Bloem B.R. (2020). The impact of the COVID-19 pandemic on Parkinson’s disease: Hidden sorrows and emerging opportunities. J. Parkinsons. Dis..

[31] Guler O., Haseki M.I. (2021). Positive psychological impacts of cooking during the COVID-19 lockdown period: A qualitative study. Front. Psychol..

[32] Boulos C., Salameh P., Barberger-Gateau P. (2017). Social isolation and risk for malnutrition among older people. Geriatr. Gerontol. Int..

[33] Ellis A., Jung S.E., Palmer F., Shahan M. (2022). Individual and interpersonal factors affecting dietary intake of community-dwelling older adults during the COVID-19 pandemic. Public Health Nutr..

[34] Kriaucioniene V., Bagdonaviciene L., Rodríguez-Pérez C., Petkeviciene J. (2020). Associations between changes in health behaviours and body weight during the COVID-19 quarantine in Lithuania: The Lithuanian COVIDiet study. Nutrients.

[35] Tribst A.A.L., Tramontt C.R., Baraldi L.G. (2021). Factors associated with diet changes during the COVID-19 pandemic period in Brazilian adults: Time, skills, habits, feelings and beliefs. Appetite.

[36] Giacalone D., Frøst M.B., Rodríguez-Pérez C. (2020). Reported changes in dietary habits during the COVID-19 lockdown in the Danish population: The Danish COVIDiet study. Front. Nutr..

[37] Rodríguez-Pérez C., Molina-Montes E., Verardo V., Artacho R., García-Villanova B., Guerra-Hernández E.J., Ruíz-López M.D. (2020). Changes in dietary behaviours during the COVID-19 outbreak confinement in the Spanish COVIDiet study. Nutrients.

[38] Sulejmani E., Hyseni A., Xhabiri G., Rodríguez-Pérez C. (2021). Relationship in dietary habits variations during COVID-19 lockdown in Kosovo: The COVIDiet study. Appetite.

[39] Changes in Dietary Behaviours During the COVID-19 Outbreak Confinement in the Adult Population (COVIDiet_Int). ClinicalTrials.gov. https://clinicaltrials.gov/ct2/show/NCT04449731?term=COVIDiet+Study&draw=2&rank=1.2020.

[40] Visser M., Schaap L.A., Wijnhoven H.A.H. (2020). Self-reported impact of the COVID-19 pandemic on nutrition and physical activity behaviour in Dutch older adults living independently. Nutrients.

[41] HEI Scores for Americans. U.S. Department of Agriculture. https://www.fns.usda.gov/hei-scores-americans.

[42] Archibald M.M., Ambagtsheer R.C., Casey M.G., Lawless M. (2019). Using Zoom videoconferencing for qualiatative data collection: Perceptions and experiences of researchers and participants. Int. J. Qual. Methods.

