# Supplementary material for: A Mixed Methods Exploration of the Impact of the COVID-19 Pandemic on Food-Related Activities and Diet Quality in People with Parkinson Disease
Source: Int J Environ Res Public Health. 2022 Sep 17;19(18):11741. doi: 10.3390/ijerph191811741 (PMC9517133; doi:10.3390/ijerph191811741)
Supplement: Supplementary file 1 [file ijerph-19-11741-s001.zip › ijerph-1876895-supplementary.pdf]

Supplemental Table S1. HEI-2015 components, point values, and standards for scoring [10,28,29].

Component	Maximum Points	Standard for maximum score	Standard for minimum score of zero
**Adequacy**			
Total fruits	5	≥0.8 c eq/1,000 kcal	No fruit
Whole Fruits	5	≥0.4 c eq/1,000 kcal	No whole fruit
Total Vegetables	5	≥1.1 c eq/1,000 kcal	No vegetables
Greens and Beans	5	≥0.2 c eq/1,000 kcal	No dark green vegetables or beans or peas
Whole Grains	10	≥1.5 oz eq/1,000 kcal	No whole grains
Dairy	10	≥1.3 c eq/1,000 kcal	No dairy
Total Protein Foods	5	≥2.5 oz eq/1,000 kcal	No protein foods
Seafood and Plant Proteins	5	≥0.8 c eq/1,000 kcal	No seafood or plant proteins
Fatty Acids	10	(PUFAS+MUFAS)/SFA >2.5	(PUFAs+MUFAs)/SFA≤1.2
**Moderation**			
Refined Grains	10	≤1.8 oz eq/1,000 kcal	≥4.3 oz eq/1,000 kcal
Sodium	10	≤1.1 g/1,000 kcal	≥2.0 g/1,000 kcal
Added Sugars	10	≤6.5% of energy	≥26% of energy
Saturated Fats	10	≤8% of energy	≥16% of energy
**Total score**	**100**		

C, cup, eq, equivalents, HEI, Healthy Eating Index, kcal, kilocalorie, MUFAs, monounsaturated fatty acids, PUFAs, polyunsaturated fatty acids, SFA, saturated fatty acids

Supplemental Table S2. Mediterranean diet score components, point values, and standards for scoring [10,28,29].

	**Mediterranean (s/week)**					
**Score**	**0**	**1**	**2**	**3**	**4**	**5**
Non-refined cereals (whole grain, bread, pasta, rice, etc.)	Never	1-6	7-12	13-18	19-31	>32
Potatoes	Never	1-4	5-8	9-12	13-18	>18
Fruits	Never	1-4	5-8	9-15	16-21	>22
Vegetables	Never	1-6	7-12	13-20	21-32	>33
Legumes	Never	<1	1-2	3-4	5-6	>6
Fish	Never	<1	1-2	3-4	5-6	>6
Red meat and products	>10	8-10	6-7	4-5	2-3	≤1
Poultry	>10	9-10	7-8	5-6	4-5	≤3
Full fat dairy products	>30	29-30	21-28	16-20	11-15	≤10
Use of olive oil in cooking (times/week)	Never	Rare	<1	1-3	3-5	Daily
Alcoholic beverages (mL/d, 100 mL= 12g ethanol	>700 or 0	600	500	400	300	<300
**Total Score**						**55**

d, day, s, servings, mL, milliliters

Supplemental Table S3. MIND diet score components, point values, and standards for scoring [10,28,29].

		MIND score	
**Score**	**0**	**0.5**	**1**
Green leafy vegetables	≤2 s/wk	>2 to <6/wk	≥6 s/wk
Other vegetables	<5 s/wk	5 to <7/wk	≥1 s/d
Berries	<1 s/wk	1/wk	≥2 s/wk
Nuts	<1/mo	1/mo or <5/wk	≥5 s/wk
Olive oil	Not primary oil		Primary oil used
Butter, margarine	>2 T/d	1-2/d	<1 T/d
Cheese	7+ s/week	1-6/wk	<1 s/wk
Whole grains	<1 s/d	1-2/d	≥3 s/d
Fish (not fried)	Rarely	1-3/mo	≥1 meal/wk
Beans	<1 meal/wk	1-3/wk	>3 meal/wk
Poultry (not fried)	<1 meal/wk	1/wk	≥2 meal/wk
Red meat and products	7+ meal/wk	4-6/wk	<4 meal/wk
Fast fried foods	4+ times/wk	1-3/wk	<1 times/wk
Pastries and sweets	7+ s/week	5-6/wk	<5 s/wk
Wine	>1 glass/d or never	1/mo-6/wk	1 glass/d
**Total score**			**15**

d, day, MIND, Mediterranean-DASH Intervention for Neurodegenerative delay, mo, month, s, servings, T, tablespoon w, week
